# Lack of Pupil Reflex and Loss of Consciousness Predict 30-Day Neurological Sequelae in Patients with Carbon Monoxide Poisoning

**DOI:** 10.1371/journal.pone.0119126

**Published:** 2015-03-04

**Authors:** Jian-Fang Zou, Qiming Guo, Hua Shao, Bin Li, Yuxiu Du, Maofeng Liu, Fengling Liu, Lixin Dai, Hung-Jung Lin, Shih-Bin Su, How-Ran Guo, Chien-Cheng Huang

**Affiliations:** 1 Clinical Division of Occupational Medicine, Institute of Occupational Health and Occupational Medicine, Academy of Medical Science, Shandong Province, China; 2 Division of Toxicology, National Institute of Occupational Health and Poison Control, Beijing, China; 3 Department of Medicine, Second People’s Hospital of Dongying, Shandong Province, China; 4 Department of Medicine, Qilu Petrochemical Corporation Hospital, Shandong Province, China; 5 Department of Medicine, Second People’s Hospital of Kenli, Shandong Province, China; 6 Department of Emergency Medicine, Chi-Mei Medical Center, Tainan, Taiwan; 7 Department of Biotechnology, Southern Taiwan University of Science and Technology, Tainan, Taiwan; 8 Department of Emergency Medicine, Taipei Medical University, Taipei, Taiwan; 9 Department of Occupational Medicine, Chi-Mei Medical Center, Tainan, Taiwan; 10 Department of Leisure, Recreation, and Tourism Management, Southern Taiwan University of Science and Technology, Tainan, Taiwan; 11 Department of Medical Research, Chi Mei Medical Center, Liouying, Tainan, Taiwan; 12 Department of Environmental and Occupational Health, College of Medicine, National Cheng Kung University, Tainan, Taiwan; 13 Department of Occupational and Environmental Medicine, National Cheng Kung University Hospital, Tainan, Taiwan; 14 Department of Child Care and Education, Southern Taiwan University of Science and Technology, Tainan, Taiwan; 15 Department of Emergency Medicine, Kuo General Hospital, Tainan, Taiwan; Institute for Health & the Environment, UNITED STATES

## Abstract

**Background:**

Predicting the neurological sequelae of carbon monoxide poisoning (COP) has not been well studied. We investigated the independent predictors of neurological sequelae in patients with COP and combined these predictors to predict the prognosis.

**Methods:**

This study was conducted at four hospitals in Shandong Province, China. Data were retrospectively collected from 258 patients with COP between November 1990 and October 2011. Thirty-day neurological sequelae were the primary endpoints.

**Results:**

A lack of pupil reflex and a loss of consciousness appear to be independent predictors for neurological sequelae in patients with COP. The presence of either one had a sensitivity of 77.0% (95% confidence interval [CI]: 69.3–83.2), a specificity of 47.1% (95% CI: 38.3–56.0), positive predictive value (PPV) of 62.9% (95% CI: 55.2–70.1), and a negative predictive value (NPV) of 63.6% (95% CI: 52.6–73.4). With both predictors present, the sensitivity was 11.5% (95% CI: 6.9 to 18.3), the specificity was 99.2 (95% CI: 94.7–100.0), the PPV was 94.1% (95% CI: 69.2–99.7), and the NPV was 49.0% (95% CI: 42.5–55.5).

**Conclusions:**

The risk for neurological sequelae apparently increased with the number of independent predictors. In patients with both predictors, the risk for neurological sequelae was 94.1%. Almost all (99.2%) patients with neither predictor had no neurological sequelae. This finding may help physicians make decisions about and dispositions for patients with COP. For patients with a higher risk, earlier treatment and more appropriate utilization of health care services, including hyperbaric oxygen, should be considered.

## Introduction

Carbon monoxide poisoning (COP) results in an estimated 50,000 emergency department visits in the United States annually and is one of the leading causes of poisoning death [1]. However, because COP is commonly misdiagnosed, the true numbers are likely to be much higher [2]. Unfavorable neurological sequelae can occur immediately after exposure and can either persist or be delayed, but they generally occur within 20 days after COP [3]. Neurological sequelae lasting one month or more appear to occur in 25 to 50 percent of patients with a loss of consciousness or with carboxyhemoglobin levels greater than 25 percent [3]. The recommended treatment for acute COP is 100 percent normobaric oxygen, commonly delivered from a reservoir through a face mask that prevents rebreathing [3]. Hyperbaric-oxygen therapy is often recommended for patients with acute COP, especially if they have lost consciousness or have severe poisoning [3].

A double-blind randomized study [3] reported that COP patients given three hyperbaric oxygen treatments within 24 hours of presentation manifested approximately one-half the rate of neurological sequelae at 6 weeks, 6 months, and 12 months after treatment than did those treated with normobaric oxygen. Bed rest and avoiding stressful procedures for the first 10 days after any prolonged hypoxic event may also lower the risk [4]. Gradual recovery over 3 to 12 months is common, but impaired attention or executive function, Parkinsonism, or signs of corticospinal tract damage can persist. Anticipating and recognizing the high risk of neurological sequelae should lead to earlier and more appropriate utilization of health care Services, including hyperbaric oxygen [4]. However, hyperbaric oxygen has many limitations, such as relative inconvenience and high cost, and the complications of hyperoxic seizures, aural barotrauma, anxiety, and oxidative stress [3]. Selecting high-risk patients who would most likely benefit is more appropriate. Recognizing the high risk of neurological sequelae can also help physicians arrive at a prognosis. However, the accuracy of predictions of the neurological sequelae of COP are inconsistent and contradictory; thus, prediction seems impractical. One study [5] proposed that elderly patients with more comorbidities, but shorter lucid intervals and fewer dangerous activities of daily living, are more likely to have a poor prognosis. However, “more complications, lucid intervals, and dangerous activities of daily living” are difficult to define. Another study [6] proposed that being exposed to carbon monoxide for 24 hours or more is an independent risk factor for neurological sequelae. In clinical practice, it is usually not possible to determine the length of exposure because patients almost always lose consciousness without a witness. Others recommend that a patient with a carboxyhemoglobin (COHb) level exceeding 25 percent [7,8] or 40 percent [9], regardless of signs or symptoms, should be considered at high risk for neurological sequelae and should be treated with hyperbaric oxygen. However, COHb blood tests are not available in every hospital. Moreover, because there is a delay before many patients arrive at a hospital for treatment, the COHb level may no longer be useful. To clarify this issue, we explored independent mortality predictors in patients with COP in our clinical settings.

## Methods

### Study design, setting, study population, and selection of participants

Data were retrospectively collected from patients with COP at four hospitals in Shandong Province, China: the Institute of Occupational Health and Occupational Medicine, Second People’s Hospital of Dongying, Qilu Petrochemical Corporation Hospital, and Second People’s Hospital of Kenli between November 1990 and October 2011. Patients were enrolled if they had documented exposure to carbon monoxide (elevation of the COHb level or the ambient carbon monoxide concentration) or obvious exposure to carbon monoxide, and if they had any of the following symptoms: loss of consciousness, confusion, headache, malaise, fatigue, forgetfulness, dizziness, visual disturbances, nausea, vomiting, cardiac ischemia, or metabolic acidosis (a calculated base excess lower than-2.0 mmol per liter or a lactate concentration higher than 2.5 mmol per liter). If the COHb level was below 10 percent, the patient was eligible only if COP was the only plausible diagnosis. Patients were excluded if they had a history of neurological disease or psychiatric disorders.

### Data Collection and Definition of Variables

All the patients were given with 100 percent oxygen at the time that COP was suspected. The protocol of hyperbaric oxygen therapy was that patients were placed in a multiplace chamber pressurized with compressed air; treating pressure was 0.2 MPa (2 ATA) with 100% O2 through a face mask or endotracheal tube for 120 minutes, once a day. The treatment period was depended on the individual condition of the patients. The Institutional Review Boards (IRBs) of Academy of Medical Science of Shandong Province approved the study protocol. The IRBs waived the need for informed consents (written and oral) from the participants because this is an observational study. Patient records/information was anonymized and de-identified prior to analysis. The reviewers were blinded to the patients’ hospital course and outcomes. Information for a number of demographic and clinical variables for each patient was recorded. Any variable not present in the patient’s medical history or physical exam was considered absent.

*Elderly* was defined as ≥ 65 years old. We used an age variable of > 35 years old based on a study [6] which reported that being > 35 years old was a risk factor for neurological sequelae. *Altered mental status* was defined as any state of awareness at admission that differed from the normal awareness of a conscious person. *Loss of consciousness* was defined as a transient loss of consciousness [2]. The *lack of a pupil reflex* was defined as a lack of response to light stimulation in one eye.

Overall, 303 patients met the criteria of COP. Two hundred and fifty eight patients were enrolled after excluding patients who had a history of neurological or psychiatric disorders or insufficient data. All the 258 patients enrolled into this study were all seen on day 30 post-poisoning. The enrolled patients were divided into two groups: [i] without 30-day neurological sequelae and [ii] with 30-day neurological sequelae. All the study variables were used for comparisons between the two groups.

### Definition of Endpoint

**Fig 1:**
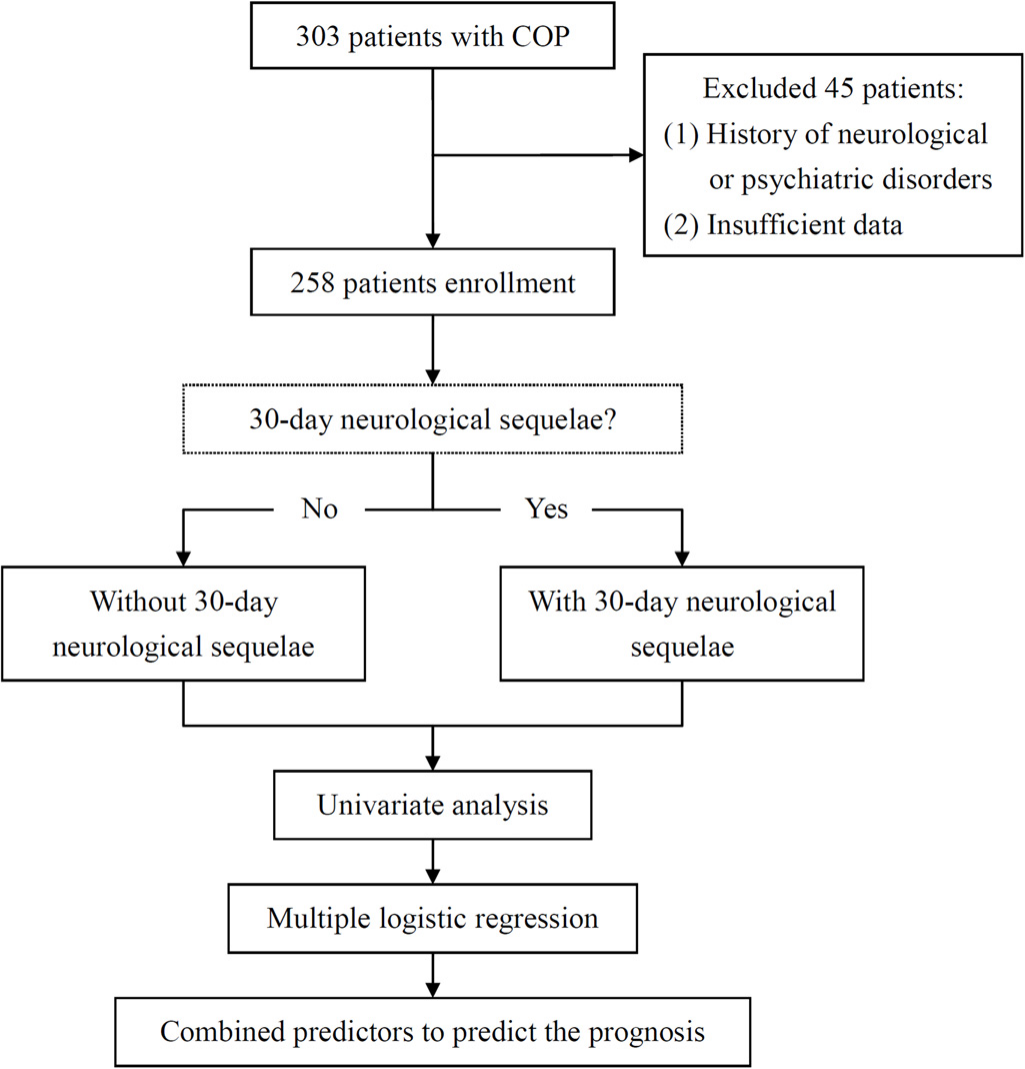
Patient enrollment and assignment to the without 30-day neurological sequelae or with 30-day neurological sequelae along with the flow of how the prediction was developed. COP, carbon monoxide poisoning.

We used the 30-day neurological outcome as the primary endpoint. Patients who had any neurological sequelae on neurological examination by neurologist 30 days after COP (memory loss, impairments of concentration or language, inability to calculate, affective changes, vestibular problem, Parkinsonism, or corticospinal tract signs of damage, etc.) [9,10] were considered ‘‘with 30-day neurological sequelae” for this analysis. Fig. 1 showed the study flowchart.

### Data analysis

All analyses were done using SPSS 16.0 for Windows (SPSS Inc., Chicago, IL, USA). Continuous data are means ± standard deviation (SD). Comparisons between the two groups were made using either an independent-samples *t* test (assuming a normal distribution) or Mann-Whitney-Wilcoxon tests (assuming a non-normal distribution) for the continuous variables. Either a χ^2^ test or a Fisher’s exact test was used for categorical variables. The significant α level was set at 0.1 for univariate variables that were included in a multiple logistic regression analysis of risk for 30-day neurological sequelae. Significance was set at *p* < 0.05 (two tailed).

## Results

The final study cohort consisted of 258 patients (123 men [48%] and 135 women [52%]) (Table 1). Their ages ranged from 6 to 97 years (mean age: 55 ± 22; median: 59). One hundred thirty-nine patients (53.9%) had 30-day neurological sequelae. Two hundred patients (77.5%) had undergone hyperbaric oxygen therapy.

**Table 1:** Univariate analysis of variables of 258 patients with carbon monoxide poisoning.

	30-Day Neurological Sequelae			
	Without	With	All	
Variable	(n = 119)	(n = 139)	(n = 258)	*p*-value
Age (years)*	51 ± 22	58 ± 22	55 ± 22	0.013
> 35 (%)	76.5	82.0	79.5	0.272
≥ 65 (%)	29.4	37.4	33.7	0.176
Gender: Male (%)	44.8	50.8	48.0	0.351
Systolic blood pressure (mmHg)*	129.4 ± 19.0	130.3 ± 24.6	129.9 ± 22.1	0.743
Heart Rate (1/min)*	87.2 ± 16.5	87.2 ± 17.3	87.2 ± 16.9	0.974
Respiratory rate (1/min)*	19.9 ± 1.9	20.6 ± 2.7	20.3 ± 2.4	0.018
Body temperature (°C)*	36.6 ± 0.6	36.6 ± 0.6	36.6 ± 0.6	0.359
Occupational exposure (%)	9.2	16.5	13.2	0.084
Current smoker (%)	12.6	12.9	12.8	1.000
Hypertension (%)	18.5	20.9	19.8	0.633
Diabetes (%)	6.7	2.1	4.3	0.119
Altered mental status (%)	29.4	40.3	35.3	0.068
Lack of pupil reflex (%)	4.2	15.1	10.1	0.004
Loss of consciousness (%)	49.6	73.4	62.4	< 0.001
Headache (%)	49.6	33.1	40.7	0.008
Dizziness (%)	66.4	47.5	56.2	0.002
Nausea/vomiting (%)	32.8	41.0	37.2	0.197
Positive Babinski’s reflex (%)	8.4	18.0	13.6	0.025
Urinary incontinence (%)	19.3	28.8	24.4	0.078
Hyperbaric oxygen therapy (%)	68.6	87.5	78.7	< 0.001

*mean ± standard deviation (SD).

Univariate analysis showed that patients with the following variables had a higher risk for neurological sequelae (*p* < 0.1): older age, higher respiratory rate, occupational exposure, altered mental status, lack of pupil reflex, loss of consciousness, less headache, dizziness, positive Babinski’s reflex, urinary incontinence, and hyperbaric oxygen therapy (Table 1). Multiple logistic regression modeling, using the results of the univariate comparison with *p* < 0.1 (Table 1), showed that the presenting variables independently associated with 30-day neurological sequelae were a lack of pupil reflex and a loss of consciousness (Table 2).

**Table 2:** Multivariate logistic regression model using the results of the univariate comparison with *p*-value < 0.1 of 258 patients with carbon monoxide poisoning

	Odds Ratio (95% Confidence Interval)		
Variable	Full Model	Final Model	*p*-value
Age	1.0 (0.9–1.0)	NA	
Respiratory rate	1.0 (0.9–1.2)	NA	
Occupational exposure	0.8 (0.3–2.5)	NA	
Altered mental status	0.74 (0.4–1.6)	NA	
Lack of pupil reflex	3.2 (1.0–10.3)	3.1 (1.0–9.2)	0.043
Loss of consciousness	2.1 (1.0–4.1)	2.3 (1.2–4.4)	0.008
Headache	0.8 (0.3–1.9)	NA	
Dizziness	0.6 (0.3–1.3)	NA	
Positive Babinski’s reflex	1.2 (0.5–2.9)	NA	
Urinary incontinence	1.0 (0.5–2.2)	NA	

NA: not available; variable not included in the final model.

The presence of either of the two predictors had a sensitivity of 77.0% (95% confidence interval [CI]: 69.3–83.2), specificity of 47.1% (95% CI: 38.3–56.0), positive predictive value (PPV) of 62.9% (95% CI: 55.2–70.1), and negative predictive value (NPV) of 63.6% (95% CI: 52.6–73.4). With both predictors present, the sensitivity was 11.5% (95% CI: 6.9–18.3), the specificity was 99.2 (95% CI: 94.7–100.0), the PPV was 94.1% (95% CI: 69.2–99.7), and the NPV was 49.0% (95% CI: 42.5–55.5) (Table 3).

**Table 3:** Sensitivity, specificity, positive predictive value (PPV), and negative predictive value (NPV) of the number of independent predictors for 30-day neurological sequelae in patients with carbon monoxide poisoning.

Number of Variables	Sensitivity	Specificity	PPV	NPV
1	77.0 (107/139)	47.1 (56/119)	62.9 (107/170)	63.6 (56/88)
2	11.5 (16/139)	99.2 (118/119)	94.1 (16/17)	49.0 (118/241)

All data are % (number).

## Discussion

We found that the lack of a pupil reflex and a loss of consciousness were independent predictors for 30-day neurological sequelae in patients with COP. In addition, these two predictors are memorable, immediately available, and applicable in clinical practice. The risk for 30-day neurological sequelae apparently increased with the number of independent predictors. In patients with both predictors, the risk for neurological sequelae was 94.1%. In patients with neither predictor, 99.2% had no 30-day neurological sequelae. This finding may help physicians make decisions about and dispositions for patients with COP. In patients with a higher risk for 30-day neurological sequelae, earlier treatment and more appropriate utilization of health care services, including hyperbaric oxygen, should be considered.

With an extremely high specificity (99.2%, missing only 1 in 119 cases), the main use of our study results is to exclude patients without neurological sequelae, not for screening because of its low sensitivity (11%). With a high incidence of neurological sequelae in patients with COP (53.9% in 30 days), in settings with limited neurology support, physicians will find it helpful to allocate the resources. In setting where a sensitivity is desirable, physicians can use a single predictor (with 77.0% sensitivity) to screen patients for neurological sequelae, but that is not a strength of this study.

The lack of a pupillary reflex or an abnormal pupillary reflex can be caused by optic nerve damage, oculomotor nerve damage, brain stem damage, or depressant drugs, such as barbiturates [11]. However, in patients with COP, the lack of a pupillary reflex indicates brainstem injury [12]. Acute brain injury in CO-exposed patients appears to arise primarily from hypoxia [13]. Studies with mice [14], however, have shown that cerebral blood flow initially increases within minutes of CO exposure. Blood flow remains elevated until consciousness is lost, and then transient cardiac compromise causes blood pressure to decrease. Because of this, autoregulation until cardiovascular homeostasis is exhausted and asphyxia or apnea begins; brain hypoxia is probably not an initial feature of COP [15]. Neurons are the central nervous system cells most vulnerable to hypoxic—ischemic insult, and they have the highest oxygen and glucose demands [16]. Acute and intense COP can lead directly to diffuse hypoxic—ischemic encephalopathy predominantly involving the gray matter [16]. The globus pallidus is the most common site of involvement in COP [12]. The caudate nucleus, putamen, and thalamus are occasionally involved in COP, but less often than is the globus pallidus [16]. Involvement of the brainstem may be a reflection of more severe poisoning because the posterior structures are more resistant to hypoxia [12].

Loss of consciousness is a commonly accepted indication for hyperbaric oxygen therapy [1]. Intubation with mechanical ventilation should be considered in those with loss of consciousness [3]. Consciousness requires normal activity of the cerebral cortex because the hemispheres are the substrate for awareness of the self and the environment, and they embody the sentient functions that define human intellectual existence [17]. Therefore, anything that diffusely depresses the activity of cerebral cortical neurons will produce a loss of consciousness [18]. In general, this can be due to actual destruction of cortical neurons or to conditions that suppress the activity of the cortex, which is generically called “encephalopathy” [18]. However, this normal activity of the cerebral cortex also requires normal activity in the reticular formation of the rostral brain stem, which extends from the mid pons through the diencephalon and is called the reticular activating system [19]. The reticular activating system can function normally even after the cerebral cortex has been destroyed (e.g., after diffuse cerebral anoxia caused by cardiac arrest) [13]. In general, the reticular activating system and brain stem are more resistant to damage than is the cerebral cortex [19]. In the case of a patient with diffuse cerebral cortical damage, the surviving reticular activating system and brain stem may be capable of supporting a crude sleep-waking vegetative state [19,20]. In contrast, the cerebral hemispheres cannot function without reticular activation [19,20]. Bilateral loss of the reticular activating system at the midbrain level (for example from ischemic or hemorrhagic transection of the upper brain stem), will terminate all sentient cerebral activity [19,20]. Carbon monoxide also stimulates guanylyl cyclase, which upregulates cyclic guanosine monophosphate and causes cerebral vasodilation; this has been associated with the loss of consciousness in an animal model of COP [21]. The role of nitric oxide (NO) and other oxygen free radicals has been researched extensively in the setting of COP. Many animal studies have reported that exposure to carbon monoxide causes cerebral vasodilation, which leads to a loss of consciousness and increased NO levels [21].

This study has several limitations. First, we did not analyze the duration of exposure to carbon monoxide or the level of COHb, because these data were unavailable for all but a few patients. However, the diagnosis of COP cannot depend solely on the COHb level, because COHb testing is not available in every hospital. Moreover, many patients arrived at the hospital after a delay, which resulted in a poor correlation between the COHb level and the patient’s prognosis. In addition, many studies [1,3,5,6] report that the COHb level is not a predictor for 30-day neurological sequelae. The length of time that a patient was exposed to carbon monoxide is usually unavailable and incorrect because the victim may be confused or may have lost consciousness. Second, data were collected from a retrospective chart review. The records of these clinical presentations may not have been completely documented. Third, there are no data on the potential for toxic co-ingestions, which might alter the initial neurological examination. Fourth, the number of patients might have been too small to generate sufficient statistical power; therefore, future studies with larger study populations are warranted. Fifth, severity of injury and concomitant inhalation injury were not recorded. Further studies about these two variables are needed because they may have significant role for predicting neurological sequelae.
